# Could Mucosal TNF Transcript as a Biomarker Candidate Help Optimize Anti-TNF Biological Therapy in Patients With Ulcerative Colitis?

**DOI:** 10.3389/fimmu.2022.881112

**Published:** 2022-05-19

**Authors:** Guanglin Cui, Jon Florholmen, Rasmus Goll

**Affiliations:** ^1^Research Group of Gastrointestinal Diseases, The Second Affiliated Hospital of Zhengzhou University, Zhengzhou, China; ^2^Faculty of Health Science, Nord University, Campus Levanger, Levanger, Norway; ^3^Division of Gastroenterology, Department of Internal Medicine, University Hospital of North Norway, Tromsø, Norway

**Keywords:** mucosa, tumor necrosis factor, inflammatory bowel disease, anti-TNF therapy, biomarker

## Abstract

Anti-tumor necrosis factor (TNF) biological therapy has generally been accepted as a standard therapeutic option in inflammatory bowel disease (IBD) patient who are refractory to steroids or immunomodulators. However, the primary and secondary nonresponse rates to anti-TNF bioagents in patients with IBD are high. To improve the response rate, anti-TNF bioagents must be offered to the appropriate IBD patients, and the withdrawal of anti-TNF bioagents needs to be done at the right time. In this context, reliable and reproducible biomarkers can provide important supportive information for clinicians to make correct decisions based on the patient’s individual situation. In this review, we summarized the current understanding of using mucosal TNF transcript (*TNF*) to improve the precision of anti-TNF biological therapy strategies in patients with ulcerative colitis (UC). Analysis of published literature showed that mucosal *TNF* could affect the precision of the early identification of candidates who will benefit from anti-TNF therapy prior to treatment, the assessment of response and mucosal healing, and the prediction of discontinuation of anti-TNF biological therapy and relapse after drug withdrawal. Challenges and limitations of using mucosal *TNF* as a biomarker in applying individualized anti-TNF biological therapy in patients with UC still remain and need to be further investigated.

## Introduction

Ulcerative colitis (UC), that is one of main types of inflammatory bowel disease (IBD), is a group of chronic inflammatory disorders that mainly affect the colorectal tract. Although the exact etiology for UC has not been fully identified thus far, the currently most cited hypothesis proposes that a combination of environmental factors, genetic predisposition, and dysregulated immune response may significantly increase the risk for UC (1). Recent epidemiological evidence has highlighted an increasing incidence of IBD worldwide, and many developing countries previously thought of as traditionally low-incidence regions have been now reported to have a dramatically increasing rate of UC following the acceleration of industrialization and urbanization (2–7).

The traditional approaches for UC management rely on diverse medicines including aminosalicylates, corticosteroids and immunomodulators according to disease activity and severity. The clinical goals of treatments are to suppress inflammation, at best obtain mucosal healing, and finally improve the UC patient’s quality of life. However, being refractory to existing medicines and disease relapse have frequently been reported (8, 9). In the case of drug- refractory disease relapse, alternative second line therapeutic strategies are currently used (10, 11). Currently, neutralizing TNF monoclonal antibodies have been widely accepted as one of the standard strategies for the treatment of severe steroid or immunomodulator-refractory or -dependent IBD patients (12), which results in remarkably improved rates of disease remission and mucosal healing (12). Even so, not all IBD patients respond well to anti-TNF therapy (13); the primary response rate to the initial anti-TNF treatment is only ~60% and 20~30% of these responders will stop responding at some point during maintenance therapy (13–17). Furthermore, more than 30% of IBD patients in remission during the first year and over 60% of IBD patients in remission within 5 years will relapse after withdrawal of anti-TNF bioagents (13, 18, 19). Finally, escalating costs and several site effects are pertinent considerations with applications of anti-TNF biological therapy in patients with IBD (20–22). To reduce the incidence of primary and secondary nonresponse and improve its efficiency in patients with IBD, it is highly clinically and economically relevant to offer anti-TNF bioagents to IBD patients who will benefit from anti-TNF biological therapy given their individual condition (23–29).

Currently, the clinical outcomes of anti-TNF biological therapy in patients with UC are mainly defined based on validated endoscopic or histologic evaluations. However, to identify the responders and non-responders pretreatment and to predict relapse after remission, effective biomarkers can provide useful and critical information for understanding and analyzing disease activity, therapeutic response, and relapse of UC (30–34). As we know, colonoscopy is the “gold standard” tool for monitoring mucosal healing and is regularly performed in IBD patients with anti-TNF therapy. In addition, colonoscopy allows the collection of colorectal mucosal samples from the inflamed site of UC patients for histopathological evaluation of disease activity and changes, in conjunction with clinical indices, the response to medicines can be assessed and monitored (9, 35–38). As TNF plays an essential role in the pathogenesis of UC and one of the main working mechanisms for anti-TNF biologic agents in suppressing colorectal inflammation is to neutralize and decrease TNF levels in UC inflamed tissues (39); therefore, combined with histological and colonoscopy observations, TNF level changes in the inflamed mucosa could be considered an index of the efficacy of anti-TNF treatment in patients with UC (40, 41). Indeed, a growing body of evidence from various studies has examined and highlighted the potential of mucosal TNF transcript (*TNF*) as a biomarker of disease activity, the time of switching from ongoing conventional treatment to anti-TNF therapy and selecting candidates, the anti-TNF therapeutic response, and timepoints for drug withdrawal and relapse after anti-TNF discontinuation in patients with UC (42–48). Therefore, we conducted this review to provide an overview of the current understanding of mucosal *TNF* as a biomarker in the context of precision medicine algorithms in UC patents undergoing anti-TNF biological therapy.

## Could Serum TNF Protein Levels Be Used as a Biomarker Candidate in UC Patients With Anti-TNF Biological Therapy?

Serological biomarkers have several advantages such as serum samples being more easily accessible, lower cost, more reproducible and more acceptable by patients than tissue biopsies (49, 50). However, the sensitivity of serological biomarkers is more easily influenced by systematic factors and sometimes lower than that of mucosal biomarkers in the reflection of inflammation and disease activity in patients with UC (23).

Regarding whether the serum TNF level could be used as a biomarker in the evaluation of disease activity in patients with UC. Owczarek et al. (51) reported that increased serum TNF levels correlated only with CD activity but not with the disease activity of UC. Avdagić et al. confirmed that serum TNF levels were not associated with disease activity in either CD or UC patients (52). However, Mateos et al. (53) recently measured the plasma concentration of TNF pretreatment in 30 active CD patients with infliximab (IFX) induction therapy and reported that increased TNFα levels were associated with an unfavorable response to IFX (53). Therefore, the reliability of a serological TNF level as a biomarker in predicting the anti-TNF response in patients with IBD is still unclear. Moreover, TNF intestinal mucosal levels were detectable in 100% of patients, while TNF serum levels were only detectable in 75% of patients (54). Therefore, the serum TNF level might not be an adequate biomarker for an assessment of disease activity in patients with UC (55, 56). One of the possible explanations is that serum TNF levels might be influenced by many factors in the body and not precisely reflect the degree of inflammation in the local mucosal environment in patients with IBD. Measuring TNF directly in the colorectal mucosa more accurately reflects the local environmental expression compared to serum levels in patients with UC (38).

Thus, the mucosal *TNF* level is a promising biomarker candidate and a potential tool for precision medicine in UC patients with anti-TNF therapy (37, 42, 43, 46, 47, 57, 58).

## The Potential Role of the Mucosal *TNF* Level as a Biomarker in Anti-TNF Candidate Selection in Patients With UC

It is well acknowledged that a reliable and powerful biomarker is important to help clinicians make decisions regarding identifying the anti-TNF candidates and the timing of anti-TNF biologic agent withdrawal.

In terms of general mechanisms, the mechanism of anti-TNF biologic agents in treating IBD is to suppress inflammation *via* neutralizing and decreasing of TNF levels and to bind with TNF receptors in the inflamed mucosa and to induce mucosal cell apoptosis. Thus, the changed expression level of mucosal TNF may directly reflect therapeutic responses, e.g., suppression of inflammation degree and changes in disease activity to different medicines (58). Ishiguro reported that an enhanced mucosal TNF level was associated with the degree of inflammation in active UC specimens (59). By using an optimized q-PCR protocol for the precise quantification of *TNF* levels in endoscopic mucosal biopsies (60, 61), our group demonstrated that increased mucosal *TNF* (messenger RNA, mRNA) levels correlated with the UC disease activity index (UCDAI) score in newly diagnosed UC patients without treatment (58). Matsuda et al. quantified the mucosal expression levels of TNF-α, interleukin (IL)-6, IL-8, and IL-10 transcripts, and confirmed that the *TNF* level was significantly increased in the colonic mucosa in patients with steroid naïve UC (62). Furthermore, our validation data demonstrated that the baseline mucosal *TNF* level was a promising biomarker in patients with UC (23). In conjunction with histological inflammatory activity scores, mucosal *TNF* level could precisely predict the need for biological therapy within the first year after the diagnosis of disease in patients with UC (23). Therefore, mucosal TNF transcript level might hold a potential for anti-TNF candidate selection.

To evaluate mucosal healing, colonoscopy is regularly performed during the anti-TNF therapy treatment period. In addition, biopsies taken by colonoscopy are critically important for histological evaluation of mucosal inflammation and disease activity changes in response to anti-TNF therapy. Therefore, the use of colonoscopic biopsy for the measurement of mucosal *TNF* is practicable and feasible in UC patients with anti-TNF therapy.

### Validation of Mucosal *TNF* as a Reliable and Reproducible Biomarker in Patients With UC

To evaluate the reliability and power of a biomarker candidate, Siegel et al. have proposed that a new biomarker must be validated before it can be used in the clinical management of IBD patients (63, 64).

Following the validation principle of a potential biomarker candidate (65, 66), we conducted a two-step procedure to validate mucosal *TNF* as a biomarker in UC patients with anti-TNF biological therapy (23). In the first step, we compared the power of this biomarker candidate with commonly used biomarkers and clinical parameters i.e., fecal calprotectin, the UC disease activity index (UCDAI), the Mayo endoscopic score and the Robarts’ histopathology index (RHI) scores in predicting the severe clinical outcome in a calibration cohort with 66 UC patients. We found that the mucosal *TNF* had the best test performance with a sensitivity, specificity, and diagnostic odds ratio (DOR) compared with the above selected biomarker and clinical parameters. In the second step (validation test), the results demonstrated that the validated cutoff values of mucosal *TNF* with histological index (TNF ≥18,000 copies, RHI ≥ 9) showed a high reliability and specificity to predict a need for anti-TNF biological therapy within the first year of disease after diagnosis in a cohort with 89 UC patients (23).

### The Role in Anti-TNF Candidate Selection in Patients With UC

**Figure 1:**
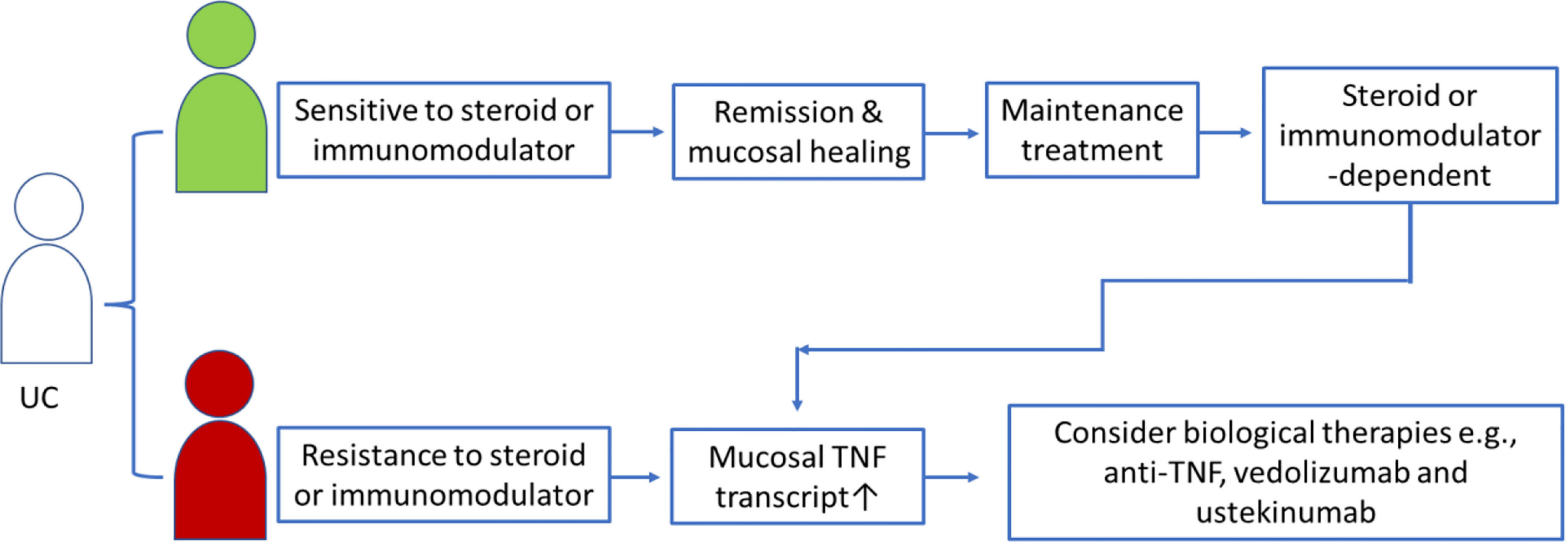
Schematic presentation of mucosal *TNF* in helping clinicians to select anti-TNF candidates in UC patients treated with steroid or immunomodulators.

In the context of precision medicine, medicines must be offered to patients who will benefit from or positively respond to medicines. To offer anti-TNF treatment to the right patients at the right time, a biomarker that can differentiate between anti-TNF-resistant and anti-TNF-sensitive IBD patients is needed (67). The strategy for clinicians for anti-TNF candidate selection in clinical practice mainly considers whether this patient is refractory to or dependent on steroids. However, such patient selection might include some unresponsive patients, and data have shown that only ~60% of IBD patients respond to the initial anti-TNF biological treatment (40, 68). A recent research has highlighted the potential for single-cell mapping tools to identify CD candidates for anti-TNF therapy (69), in which the GIMATS module that consisted a set of cellular and molecular parameters, such as IgG plasma cells, inflammatory mononuclear phagocytes, activated T cells, and stromal cells, at diagnosis could differentiate the responder and nonresponder to anti-TNF therapy in patients with CD prior to treatment (69). Other studies revealed that mucosal *TNF* levels might be a biomarker that can help clinicians identify appropriate patients and opportunities for anti-TNF therapy in patients with UC (37, 42, 43, 58). Therefore, we observed persistently high mucosal *TNF* levels in UC patients after treatment with steroids for a certain period, which might indicate a steroid-refraction/dependent phenotype and the need to consider switching to anti-TNF biological therapy (refer to **Figure 1**). On the other hand, we might prolong steroid use for a while in partial responders if the mucosal *TNF* level is nearly normalized after excluding high anti-drug antibody levels.

## Mucosal *TNF* Level Changes Predict Clinical Response to Anti-TNF Biological Therapy in Patients With UC

Matsuda et al. quantified the mucosal expression levels of TNF-α, interleukin (IL)-6, IL-8, and IL-10 transcripts, and confirmed that the *TNF* level was significantly increased in the colonic mucosa in patients with steroid naïve UC (62). Furthermore, our validation data demonstrated that the baseline mucosal *TNF* level was a promising biomarker in patients with UC (23). In conjunction with histological inflammatory activity scores, mucosal *TNF* level could precisely predict the need for biological therapy within the first year after the diagnosis of disease in patients with UC (23).

Studies have also suggested that changed mucosal *TNF* levels predict the clinical response to anti-TNF biologic agents and can be used for the evaluation of anti-TNF therapeutic efficacy. Tsukada et al. (70) have previously examined the mucosal cytokine transcript profile and its relation to disease activity in patients with UC. They reported that the *TNF* level was higher in inflamed mucosa of UC patients than in uninflamed controls. The expression level of mucosal TNF in patients with UC was remarkably decreased after prednisolone treatment (70). Raddatz et al. (71) quantitatively measured the mucosal levels of cytokine transcripts in 24 UC patients and 18 CD patients with glucocorticoid therapy. However, they found that the mucosal cytokine transcript levels were not associated with the response to a glucocorticoid therapy in either UC or CD (71). Such inconsistent results led to us to examine the potential role of mucosal *TNF* levels in the evaluation of anti-TNF therapeutic efficacy in UC patients treated with an anti-TNF bioagent (IFX). Indeed, our studies have shown that changed mucosal *TNF* levels might predict remission and mucosal healing rates in response to IFX in patients with UC (42). To investigate the correlation between the mucosal *TNF* transcript level prior to treatment and the response rate to IFX in patients with UC, we divided UC patients into 3 groups according to the cutoff values of pretreatment mucosal *TNF* values (high, medium and low mucosal *TNF* level groups) (42). We found that the rate of mucosal healing after IFX treatment in the 3 groups was very different (low vs. medium vs. high: 82% vs. 64% vs. 42%), although the UCDAI and endoscopic scores in the 3 groups before IFX treatment were comparable. Furthermore, mucosal *TNF* levels in nonremised UC patients were maintained at a higher level than those in remised patients (42). Our results indicated that pretreatment mucosal *TNF* values might have a predictive potential for the remission and mucosal healing rates in response to IFX therapy, and UC patients with a higher mucosal TNF value might need a longer period or higher dose of IFX after exclusion of high anti-drug antibody levels (42). Moreover, our recent observation data in UC patients with repeated intensified IFX induction therapy confirmed that normalization of mucosal *TNF* levels could predict long-term remission upon discontinuation of IFX (45). Other clinicians reported similar findings. Therefore, these findings provide new insights into the strengthening the use of mucosal *TNF* as a biomarker to optimize the precision of anti-TNF strategies in patients with UC. For example, to normalize the mucosal TNF level and achieve better efficacy of anti-TNF treatment, a patient with a higher *TNF* level might need a higher dose or longer duration of induction therapy with anti-TNF bioagents than patients with a lower *TNF* level.

## Could the Mucosal *TNF* Predict the Anti-TNF Biological Therapy Discontinuation and Long-Term Remission After Drug Withdrawal?

**Figure 2:**
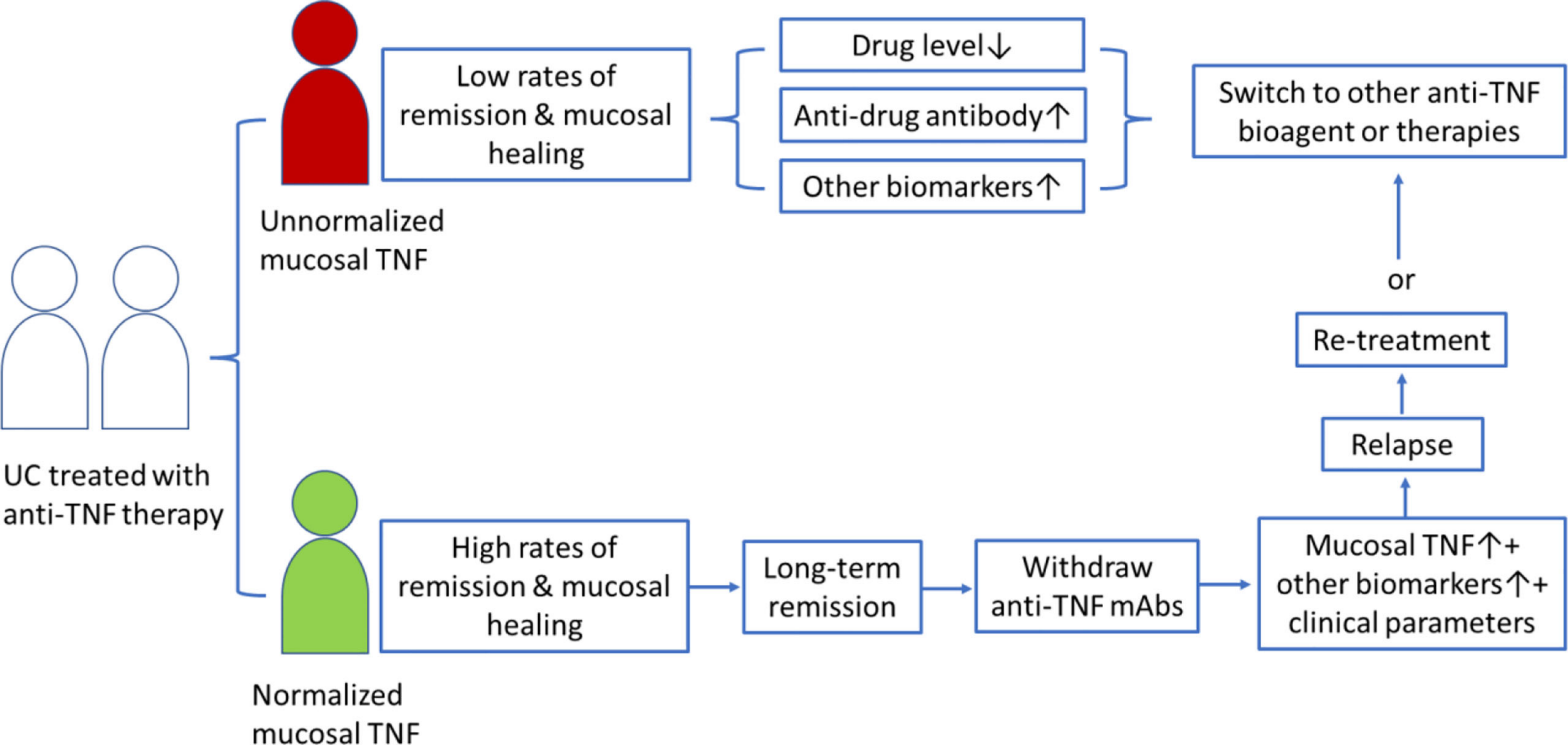
Schematic presentation of mucosal *TNF* in helping clinicians to discontinue anti-TNF therapy and predict relapse after drug withdrawal in patients with UC.

Anti-TNF biologic therapy places a heavy economic burden on patients and the health system because anti-TNF biologic agents are very expensive and have several side effects (21, 72, 73). Clinically, patients receive an insufficient treatment and a high relapse rate if the end of anti-TNF treatment is too early; in contrast, overtreatment with anti-TNF bioagents may increase side effects and costs. Therefore, the cessation of anti-TNF biologic agents must be at the right time to reduce the relapse rate after anti-TNF withdrawal (74–76). However, no consensus has been currently reached regarding the timing of anti-TNF therapy discontinuation in patients with IBD. We have previously considered ceasing anti-TNF drugs when the mucosal *TNF* level becomes normalized (44). Our data showed that a normalized mucosal *TNF* transcript level indicated a higher rate of mucosal healing during the anti-TNF treatment period and a significantly long-term remission period after IFX withdrawal in patients with UC (44). Therefore, the mucosal *TNF* level might be a predicative biomarker for long-term remission in UC patients with mucosal healing after anti-TNF drug discontinuation. Taken together, monitoring mucosal *TNF* changes in patients with UC in remission under anti-TNF biological therapy might provide useful information to predict the possibility of long-term remission after drug withdrawal (refer to the summary in **Figure 2**).

## Could Mucosal *TNF* as a Biomarker in CD Patients With Anti-TNF Therapy?

Currently, supportive evidence for using mucosal TNF transcript as a biomarker candidate was largely from UC patients (refer to **Table 2**), only a limitative studies were performed in patients with CD.

**Table 2:** Summary of predictive significance of mucosal *TNF* transcript level as a biomarker candidate in UC patients with anti-TNF therapy.

Clinical items	Predictive significance of mucosal TNF level		References
	Unnormalized	Normalized	
Patients with steroids or immunoregulators	Mucosal non-healing → swift to anti-TNF therapy	Mucosal healing → maintain treatment	(37, 42, 43, 58)
Anti-TNF therapeutic response	Symptom relief and mucosal healing rates ↓	Symptom relief and mucosal healing rates ↑	(42, 45)
Drug discontinuation	Consider to continuate or change drugs	Consider to discontinue current drug	(44)
Remission after treatment	Short-term	Long-term	(47)

For example, Schmidt et al. identified a close relationship between a decreased expression level of mucosal *TNF* and long-term remission in azathioprine or methotrexate-refractory CDs treated with infliximab (IFX) or cyclophosphamide therapies (41). Atreya et al. (77) showed that high numbers of mucosal membrane-bound TNF (mTNF) (mTNF) (+) immune cells could predict a significantly higher short-term response rate at week 12 compared to patients with low amounts of mTNF(+) cells (92% vs. 15%) in CD patents with anti-TNF therapy. Furthermore, Vatansever et al. reported that the mucosal TNF protein level before treatment determined by immunohistochemistry could predict a favorable response to anti-TNF biological therapy in 35 patients with CD (78). CD patients with high mucosal TNF protein levels at diagnosis might need earlier anti-TNF treatment (78). Schmidt et al. reported that mucosal *TNF* levels before IFX biological therapy could help to identify patients who will achieve a long-term remission in patients with CD (41). Our data confirmed that a higher mucosal *TNF* level in CD patients with mucosal healing could predict a high rate of relapse after anti-TNF drug withdrawal (47). However, the size of most of studies performed in CD patients with anti-TNF therapy were relatively small. Although this biomarker candidate has recently been validated in patients with UC (23), it is waiting to be done in a larger-scale cohort of CD patients.

## Challenges and Limitations Regarding Mucosal *TNF* as a Biomarker Candidate in UC Patients With Anti-TNF Biological Therapy

Despite encouraging findings indicating that mucosal *TNF* levels could serve as a biomarker for individualized anti-TNF therapy in patients with IBD, a number of challenges remain.

For example, how to use biomarkers to identify patients who respond to anti-TNF therapy early has become a major challenge. However, to the best of our knowledge, no comparison studies of anti-TNF therapeutic efficacy between IBD patients with a normalized and high TNF levels are currently available. The prescription of anti-TNF therapy mainly depends on the disease activity defined by clinical symptoms and endoscopic observation. Thus, it is unclear whether an IBD patient with a normalized mucosal *TNF* level can benefit from anti-TNF biological therapy. As indicated by our studies, normalized mucosal *TNF* could predict a high mucosal healing response rate to anti-TNF therapy (42) and long remission time after anti-TNF biologic agent withdrawal (44, 47). To reach a higher rate of mucosal healing or remission, whether the dose or duration of anti-TNF bioagents in those partially mucosal healing patients with a high mucosal *TNF* level after excluding high anti-drug antibody levels should be increased remains to be investigated.

This review focused only on a single biomarker (mucosal *TNF*) in patients with IBD in the context of precision medicine. Mucosal *TNF* level alone as a biomarker in precision anti-TNF biological therapy may have its shortcomings. The efficacy of anti-TNF therapy also depended on the formation of anti-drug antibodies, and a high level of anti-drug antibodies might significantly decrease the efficacy of anti-TNF bioagents. Yarur et al. (79) reported that inflamed tissue anti-TNF antibody levels combined with the endoscopic disease activity score could increase the predictive power of the response to IFX in patients with IBD. D’Haens et al. (80) analyzed and validated the significance of multiple (total 13) protein biomarkers, called the endoscopic healing index (EHI), in indicating mucosal damage and repair processes in 278 patients with CD. They reported that EHI showed a good correlation with the activity identified by endoscopy and a better performance than a single biomarker (for example, serum CRP) in reflecting the rate of mucosal healing (80).

Finally, clinical studies have shown that combination therapy with anti-TNF bioagents and immunomodulators is better than monotherapy for inducing remission or mucosal healing in patients who have failed to respond or lose response to their first anti-TNF bioagent (81–84). Currently, several novel biologic agents including ustekinumab (human monoclonal antibodies block IL-12 and IL-23), natalizumab (humanized monoclonal antibody against alpha-4 integrin) and vedolizumab (humanized monoclonal antibody against alpha-4-beta-7 integrin) have been developed for potential use in the management of IBD (85). Kopylov et al. reported that ustekinumab could be a candidate for effective therapy in CD patients who become unresponsive to anti-TNF biological therapy (86). Do the mucosal *TNF* level changes reflect clinical remission and mucosal healing in IBD patients treated with these novel biologic agents? Murate et al. (48) recently reported that a combination of high baseline serum TNF concentration with low simple endoscopic score predicts a high clinical response rate to ustekinumab treatment in patients with CD. More answers to these questions may improve the precision management of IBD with anti-TNF biological therapy.

Another limitation of this biomarker is the need for endoscopic biopsies, which does not allow for easy proactive monitoring of relapse during the remission period. Most UC patients with remission will not go to hospital to take colonoscopy examination until the release occurs and symptoms become obviously. There are a number of ways to measure TNF expressing cells in colorectal biopsies taken by colonoscopy, such as RNA transcript assay by *in situ* hybridization, TNF protein by immunohistochemistry. Intensive histological evidence suggested that TNF was widely expressed on many types of cells in the microenvironment of colorectal mucosa. Studies showed that TNF both at mRNA and protein levels were known to express in many types of cells, such as epithelial cells, Paneth cells, immune cells e.g., eosinophils, TH cells and macrophages, in the colorectal mucosa (37, 58, 87–92). In addition, TNF was expressed on intestinal lymph tissues and played a regulatory effect on the maintenance of microarchitecture and the local immune cell differentiation and function in the colorectal mucosa (93). Since the heterozygosity of TNF expressing cell source in the colorectal mucosa, the bulk *TNF* in biopsies taken from different sites of colorectal mucosa may be vary and does not have enough resolution for clinical use. For example, if biopsies sampling accidentally hit Prayer’s Patch, the massive amount of macrophages will result in extremely high levels of TNF transcript. Similar results may also be happen in biopsies with high densities of eosinophils and Paneth cells. Therefore, one of strategies that might avoid this sampling shortcoming is carefully to divide intestinal regions and established normal values according to different regions. To reduce the risk of sampling bias, the clinicians need to take multiple endoscopic biopsies from the same region.

Theoretically, protein synthesis is controlled by transcription. Previous studies have validated that mucosal cytokine/chemokine transcript profiles could reflect the degree of mucosal inflammation in patients with CD (94). However, comparison studies between mucosal *TNF* and proteins in evaluation of anti-TNF therapy in patients with IBD remain to be conducted. In addition, the anti-TNF therapeutic efficacy depends on several clinical factors such as age, disease severity, dose/duration, drug level and anti-drug level, rather than a single factor. Mucosal *TNF* changes reflect only suppressed degree of TNF in the colorectal mucosa after anti-TNF biological therapy. Clinical studies reported that anti-drug antibodies were developed in most UC patients with anti-TNF therapy, high levels of anti-drug antibody were associated with the low therapeutic efficacy in patients receiving anti-TNF therapy (95–100). Therefore. the level of anti-drug antibodies has been widely used as important biomarker for the evaluation of anti-TNF therapeutic efficacy and provided useful information to monitor response and confirm treatment decisions (99, 101, 102). Notably, anti-drug antibody combined with other biomarkers, for example drug level, could enhance the predictive significance in the evaluation of response to anti-TNF bioagents (103–106). UC patients with normalized mucosal *transcript* levels might possibly had a low anti-TNF efficacy, which made it difficult for clinicians to accurately determine whether or when the treatment decision should be shifted. In such case, the clinician needed to combine anti-drug antibody level with mucosal TNF transcript to evaluate the therapeutic response to anti-TNF treatment in patients with UC. It was time to consider to swift current anti-TNF therapeutic to another medicine when the increased level of anti-drug antibody level was observed in UC patients with anti-TNF therapy. Therefore, screening and grouping mucosal *TNF* with other biomarkers into a practice biomarker group for “anti-TNF efficacy analysis” use is necessary.

In our clinical practice, we have found that a few IBD patients with normalized mucosa *TNF* levels after withdrawal of anti-TNF biologic agents might still relapse. Considering that multiple factors, such as IL-33, participate in the complex regulatory network of intestinal inflammation in IBD (36), it is likely that the combination of mucosal *TNF* with other biomarkers and clinical parameters is needed.

## Conclusion and Future Perspectives

**Figure 3:**
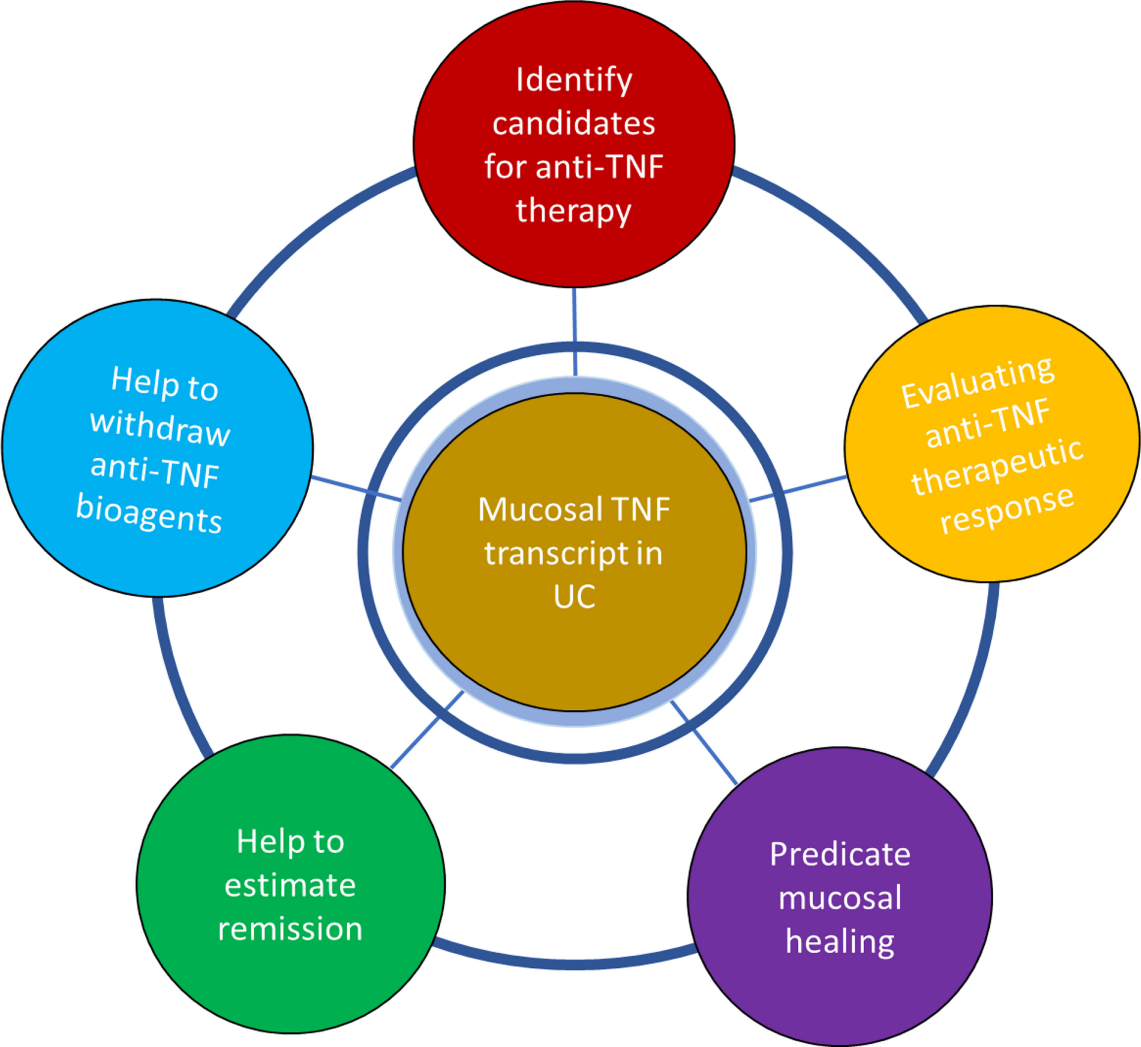
Schematic summary of the role of mucosal *TNF* in precisive/personalized anti-TNF therapy in patients with UC, incorporating integrated analysis of current published data. Analysis of current data suggested that mucosal *TNF* might play the potential role in the candidate selection for anti-TNF biological therapy, predicating mucosal healing, helping drug withdraw and long-term remission after drug withdraw.

Emerging evidence suggests that the changes in mucosal *TNF* level in IBD patients treated with anti-TNF bioagents provide translational information on the response to drugs, which is extremely important for precision anti-TNF therapy. Analysis of the current data suggested that mucosal *TNF* may potentially play a role in candidate selection for anti-TNF biological therapy, predicting remission and mucosal healing and helping predict drug withdrawal and relapse after withdrawal. Here, we summarized the clinical and translational role of mucosal *TNF* as a biomarker candidate for precision/personal treatment regimens in patients with IBD as shown in **Table 1** and **Figure 3**, incorporating integrated analysis of current data.

**Table 1:** Summary of the role of mucosal *TNF* transcript in precision strategies of anti-TNF therapy in patients with UC.

Precision strategies	Mucosal *TNF*	References
Help to select candidates	√	(37, 42, 43, 58)
Predict response	√	(42, 45)
Estimate drug discontinuation	√	(44)
Estimate long-term remission after treatment	√	(47)

However, additional studies are still needed to resolve challenges and limitations in the comprehensive use of mucosal *TNF* levels as a biomarker in precision anti-TNF therapy. For example, should we measure mucosal TNF levels before we decide to prescribe anti-TNF biologic agents to an IBD patient? Should those patients with a high TNF level be given priority consideration for anti-TNF therapy? What is the effect of anti-TNF bioagents on IBD patients with a low or normal TNF level? Answering these questions might help clinicians precisely identify candidates who respond to anti-TNF therapy. Additionally, given the complexity of the cytokine network in the colorectal mucosa, a single cytokine biomarker may not be sufficient to predict an overall response to anti-TNF therapy in all IBD patients. This challenge might be overcome by developing combination strategies involving mucosal *TNF* and other biomarkers or clinical parameters (107). Therefore, the predictive power and value of such biomarker combinations must be further tested and validated in clinical practice.

## Author Contributions

GC had the idea for this systematic review and performed the electronic search for literatures, GC and RG performed literature selection, data extraction and analysis. JF joined the data analysis and discussion. All the listed authors contributed to this manuscript in writing and final approvement.

## Funding

This study was supported by the Medical Research Program, Northern Norway Regional Health Authority (Helse Nord RHF), Norway (Program No. SFP-922-10). The funder did not play any role in paper design, data collection, data analysis, interpretation, writing of the paper. We apologize for not being able to cite all the excellent studies and reviews due to space limitations.

## Conflict of Interest

The authors declare that the research was conducted in the absence of any commercial or financial relationships that could be construed as a potential conflict of interest.

## Publisher’s Note

All claims expressed in this article are solely those of the authors and do not necessarily represent those of their affiliated organizations, or those of the publisher, the editors and the reviewers. Any product that may be evaluated in this article, or claim that may be made by its manufacturer, is not guaranteed or endorsed by the publisher.

## Abbreviations

CD, Crohn’s Disease; CDAI, Crohn’s Disease activity index; CRP, C-reactive protein; CT, cycle threshold; DOR, diagnostic odds-ratio; EHI, endoscopic healing index; ELISA, Enzyme-linked immunosorbent assay; FC, fecal calprotectin; IBD, Inflammatory bowel diseases; IFN-γ, Interferon-γ; IL, Interleukin; mRNA, Messenger RNA; q-PCR, Quantitative real-time PCR; RHI, The Robarts’ histopathology index; *TNF*, TNF transcript; TNF-α, Tumor necrosis factor-α; UC, Ulcerative colitis; USAI, Ulcerative colitis activity index.
